# Masked Delivery of Allergen in Nanoparticles Safely Attenuates Anaphylactic Response in Murine Models of Peanut Allergy

**DOI:** 10.3389/falgy.2022.829605

**Published:** 2022-02-07

**Authors:** Kevin R. Hughes, Michael N. Saunders, Jeffrey J. Landers, Katarzyna W. Janczak, Hamza Turkistani, Laila M. Rad, Stephen D. Miller, Joseph R. Podojil, Lonnie D. Shea, Jessica J. O'Konek

**Affiliations:** ^1^Department of Biomedical Engineering, University of Michigan, Ann Arbor, MI, United States; ^2^Medical Scientist Training Program, University of Michigan, Ann Arbor, MI, United States; ^3^Mary H. Weiser Food Allergy Center, Michigan Medicine, Ann Arbor, MI, United States; ^4^Department of Microbiology-Immunology, Feinberg School of Medicine, Northwestern University, Chicago, IL, United States; ^5^Department of Dermatology, Feinberg School of Medicine, Northwestern University, Chicago, IL, United States; ^6^COUR Pharmaceuticals Development Co, Inc., Northbrook, IL, United States; ^7^Department of Chemical Engineering, University of Michigan, Ann Arbor, MI, United States; ^8^Department of Surgery, University of Michigan, Ann Arbor, MI, United States

**Keywords:** nanoparticle, tolerance, food allergy, Th2, antigen-specific therapy

## Abstract

Food allergy is a growing health concern worldwide. Current allergen-specific immunotherapy (AIT) approaches require frequent dosing over extended periods of time and may induce anaphylaxis due to allergen-effector cell interactions. A critical need remains to develop novel approaches that refine AIT for the treatment of food allergies. Previous studies show that poly(lactide-co-glycolide) (PLG) nanoscale particles (NP) effectively suppress Th1- and Th17-driven immune pathologies. However, their ability to suppress the distinct Th2-polarized immune responses driving food allergy are unknown. Herein, we describe the safety and efficacy of NPs containing encapsulated peanut allergen in desensitizing murine models of peanut allergy. Peanut extract encapsulation allowed for the safe intravenous delivery of allergen relative to non-encapsulated approaches. Application of 2–3 doses, without the need for dose escalation, was sufficient to achieve prophylactic and therapeutic efficacy, which correlated with suppression of Th2-mediated disease and reduced mast cell degranulation. Efficacy was associated with strong reductions in a broad panel of Th1, Th2, and Th17 cytokines. These results demonstrate the ability of PLG NPs to suppress allergen-specific immune responses to induce a more tolerogenic phenotype, conferring protection from intragastric allergen challenge. These promising studies represent a step forward in the development of improved immunotherapies for food allergy.

## Introduction

The incidence of food allergy is increasing worldwide and currently affects ~10% of the population in the US, or more than 30 million patients (1). Clinical recommendations for food allergy primarily consist of allergen avoidance and post-exposure symptom management (2–4) Allergen-specific immunotherapy (AIT) aims for proactive treatment of food allergies, with oral (OIT), epicutaneous (EPIT), and sublingual (SLIT) immunotherapies representing potential routes of delivery (5). These therapies can temporarily desensitize patients and, in some instances, may suppress Th2 responses through the induction of regulatory and Th1 responses (6). However, OIT and other AIT strategies, which typically require daily dosing and dozens of visits, function mostly by transiently desensitizing recipients to allergen potentially requiring lifelong continuation of therapy (7–13). Further, administration of free, which we refer to as unmasked, allergen to sensitized patients bears a considerable risk of adverse reactions. In initial clinical trials, 9.4% of patients taking Palforzia, an FDA-approved OIT for peanut allergy, had an anaphylactic reaction prior to reaching maintenance dose (11) while other OIT trials have more generally resulted in significant adverse events for 22–27% of enrolled children (14). While OIT is beneficial for many patients and caregivers, technologies to induce allergen-specific tolerance safely, rapidly, effectively, and equitably remain a critical clinical need.

Previously, our group and others have demonstrated the ability to intravenously deliver protein cargo to antigen presenting cells (APCs) in the spleen and liver via poly(lactide-co-glycolide) (PLG) NPs for tolerance induction (15, 16). These APCs present the antigen on Major Histocompatibility Complexes (MHC) for recognition by T cells with minimal costimulatory signaling, which has been shown to induce antigen-specific immune tolerance in models of Th1/17-driven disease (15–18). This platform is currently under development for commercial application and has shown promise in Phase I and IIa clinical trials for celiac disease, an autoimmune disease triggered by gluten consumption (19–21). Despite the success in Th1/17 disease, food allergy represents a unique challenge. Th1/17 responses involve the secretion of pro-inflammatory cytokines (e.g., IFN-γ, IL-2, TNF-α) and cell-mediated immunity, whereas Th2 responses associated with food allergy are mediated by interleukins (e.g., IL-4, IL-5, IL-13) that activate mast cells and IgE-producing plasma cells leading to humoral immunity (22, 23). Ultimately, IgE crosslinking after allergen recognition results in histamine and other mediator release by effector cells, initiating allergic reactions. To date, no therapy has induced tolerance or demonstrated durable suppression of Th2 immune responses in food allergy (5, 8, 10, 13, 24, 25). Additionally, attempts to deliver the allergen risk anaphylaxis if not masked from immune recognition. While antihistamines, auto-injectable epinephrine, and other medications can manage consequences of the Th2 response, controlling immune activation selectively in response to allergen remains the goal.

In this report, we investigate intravenous delivery of allergen loaded NPs for their ability to attenuate allergic responses in murine models of peanut allergy. Based on our prior work and others, we hypothesize that the intravenous route may provide a potent therapeutic pathway to antigen-specific tolerance in food allergic individuals (26, 27). Intravenous delivery of unmasked peanut in pre-sensitized individuals carries significant risk of anaphylaxis (28–30). We tested whether allergen encapsulation within NPs sufficiently masks IgE binding sites to avoid anaphylaxis while enabling allergen presentation by APCs, ultimately reducing T cell activation. These studies also investigate the ability to abrogate biological outcomes associated with Th2-mediated food allergy using minimal therapeutic doses. If successful, this therapy would improve patient treatment options by reducing burden to patients and healthcare systems while potentially achieving the long-term tolerogenic effects that have largely eluded AIT to date.

## Materials and Methods

### Reagents

Peanut extract (Greer Laboratories, Lenoir, NC) was used for all intraperitoneal (i.p.) and intravenous (i.v.) immunizations and in NP formulations. For delayed type hypersensitivity (DTH) studies and for intragastric sensitization and challenge, peanut flour (Byrd Mill 12% fat, light roast) was solubilized in PBS and concentrated using a 10,000 MWCO Centricon filter unit to create a peanut extract. Aluminum hydroxide (alum; alhydrogel) was purchased from InvivoGen (San Diego, CA). Cholera toxin (CTx) from Vibrio cholera was purchased from List Biological Laboratories (Campbell, CA). PLG (50:50) with a single carboxylic acid end group and an inherent viscosity of 0.17 dL/g in hexafluoro-2-propanol was purchased from Lactel Absorbable Polymers (Essen, Germany). Poly(ethylene-alt-maleic anhydride) (PEMA) was purchased from Polyscience (Niles, IL). Grade V hen egg albumin (Ovalbumin, OVA) was purchased from Sigma Aldrich (St. Louis, MO). Cyanine5.5 amine was purchased from Lumiprobe (Cockeysville, MD). Chromogenic LAL endotoxin quantitation kit was purchased from ThermoFisher Scientific (Waltham, MA). All other reagents were purchased from Sigma Aldrich unless noted otherwise.

### Synthesis of Encapsulated [PLG(PE)] and Surface-Conjugated Peanut Extract PLG Nanoparticles (PLG-PE)

Peanut extract was encapsulated with 50:50 PLG polymer following protocols previously described (15, 16). NPs with encapsulated antigen [denoted as PLG(PE)] were synthesized using a double emulsion protocol. Briefly, 150 μL of 50 mg/mL protein (ovalbumin or peanut extract) was encapsulated in 20% w/v PLG in dichloromethane with PEMA surfactant and allowed to evaporate. NPs were washed three times by centrifugation (5,000x g, 5 min, 4°C) prior to freezing and lyophilization in a solution of 4% w/v sucrose and 3% w/v mannitol. To generate NPs containing no allergen [PLG(PBS)], solvent evaporation NPs were synthesized as described above without addition of protein and were similarly washed and lyophilized. To generate NPs with surface-conjugated allergen (denoted as PLG-PE), carbodiimide chemistry of PLG(PBS) NPs was utilized for conjugation as previously described (31). Briefly, solvent evaporation NPs were synthesized as described above without addition of protein and were similarly washed and lyophilized. Lyophilized NPs were subsequently washed three times with MilliQ water prior to incubation of NPs and 1-Ethyl-3-(3-dimethylaminopropyl)carbodiimide (EDC) at 50 and 20 mg/mL, respectively. To achieve varied loadings of surface-exposed antigen, peanut extract was incubated after activation of PLG with EDC at concentrations of 1, 2, or 4 mg/mL in PBS. To conjugate the fluorescent probe, Cyanine5.5 was incubated after activation of PLG with EDC at a concentration of 20 mg/mL in PBS (31, 32).

### Nanoparticle Characterization

The size, polydispersity, and zeta potential of the NPs were determined by dynamic light scattering (DLS) by mixing 10 μL of a 25 mg/mL NP solution into 990 μL of MilliQ water using a Malvern Zetasizer ZSP. The release of encapsulated antigen from NPs was measured over 72 h as previously described (15, 16). Briefly, 8 mg of NPs were dispersed in PBS and incubated at 37°C. At various timepoints NPs were centrifuged at 7,000 g and 4°C for 5 min and supernatant was collected and stored at 20°C for measurement using a Micro BCA assay (Pierce). Lyophilized NPs were washed by centrifugation with MilliQ water to remove cryoprotectant. Quantification of the total amount of antigen within NP was performed using the Micro BCA assay (Pierce) or CBQCA assay (ThermoFisher) as previously described by dissolving antigen-loaded NPs in 0.1M NaOH for 24 h (33).

### Mice and Immunizations

All animal procedures were approved by the University of Michigan and Northwestern University Institutional Animal Care and Use Committee and carried out National Institutes of Health Guide for the Care and Use of Laboratory Animals (NIH Publications No. 8023, revised 1978). Specific pathogen-free C3H/HeJ or Balb/c mice (female, 4–8 weeks old) were purchased from Jackson Laboratory (Bar Harbor, ME). Schedules of immunizations are shown or described in the figures and captions. Allergic sensitization was induced with intraperitoneal immunization of 20 μg of peanut extract adsorbed on 2 mg alum or by intragastric administration of 1 mg of peanut and 10 μg of CTx (34). Peanut extract alone diluted with PBS served as a control in preliminary experiments. Mice were challenged intragastrically with 20 mg of peanut on 7 alternating days during the final 2 weeks of the studies. Repeated oral challenges in mouse food allergy models increase the severity of allergic reactions, and this has been suggested to occur because SPF mice have low basal levels of goblet cells and mast cells in the intestine that increase over the course of the challenge reaching a threshold for significant reactivity after more than four oral challenges (35–38). Symptoms and body temperature were recorded every 15 min for at least 1 h after challenge and sera harvested for quantification of MCPT-1 following 7th oral challenge when reactions are maximal for all groups. For delayed type hypersensitivity (DTH) experiments, C57BL/6 mice were sensitized with 100 μL of a solution containing 1 mg/mL Peanut extract and 2 mg/mL Complete Freund's Adjuvant. The same day and 7 days post-sensitization, PLG(PE) was administered at 0, 0.05, 0.1, 0.25, 0.5, 0.75, 1, or 2.5 mg/dose. Change in pinna thickness of both ears of mice after injection of 10 μg OVA (left ear) or 10 μg Peanut extract (right ear) on Day 14 post-sensitization, and the level of increased ear swelling measured at 24 h post antigen challenge.

### Measurement of Serum Antibodies

Sera were obtained by saphenous vein bleeding. Peanut-specific IgE antibody levels were determined in serially diluted serum using peanut-coated 96-well plates and alkaline phosphatase–conjugated detection antibodies, as described previously (34). For evaluation of peanut antigen on the surface of PLG(PE) and surface-conjugated peanut extract PLG (PLG-PE) NPs, plasma from peanut-sensitized mice and anti-mouse IgG-HRP were employed as follows: in a 96-well plate, NPs were blocked at room temperature with 10% normal goat serum in trizma buffer (pH 7.35) before washing twice with excess trizma buffer. Plasma was diluted 1:1000 in trizma buffer and incubated with NPs on a rocker for 30 min at room temperature. NPs were then washed and resuspended in 1:10,000 anti-Ms IgG HRP (Thermo Fisher A16072) for 1 h, followed by additional washing. NPs were resuspended in ECL Clarity HRP substrate solution and chemiluminescent images were collected using an *in vitro* Imaging System (IVIS) after 5 min with small binning and 1 s exposure time. The flux of each well was normalized to the average flux observed from PLG(PBS) treated in this manner.

### Analysis of Cytokine Expression Recall Assays

Spleens and mesenteric lymph nodes (mLNs) were dissected and manually disrupted to generate single-cell suspensions. Red blood cells were depleted from splenocytes with ACK lysing buffer. Lymphocytes were resuspended in culture medium and plated at 800,000 cells per well in tissue culture-treated 96-well flat-bottom plates. Cells were cultured *ex vivo* with or without peanut (5 μg/mL). After 72 h, cytokine secretion was measured in cell-culture supernatants using a Luminex Multiplex detection system (Millipore, Billerica, Mass) or by ELISA (University of Michigan Cancer Center Immunology Core). For each sample, data were determined as follows:

*[Peanut stimulated] – [Unstimulated]* = *Total (pg/mL) for each cytokine (Mean of duplicate determinations)*

### Assessment of Hypersensitivity Reactions

Reactions were evaluated for at least 1 h after challenge by using the following scoring system [modified from (34, 39)]: 0, no symptoms; 1, prolonged rubbing and scratching around the nose, eyes, or head; 2, puffiness around the eyes or mouth, diarrhea, piloerection, and/or decreased activity with increased respiratory rate; 3, labored respiration, wheezing, stridor, and/or cyanosis around the mouth and tail; 4, tremor, convulsion, no activity after prodding, and/or moribund; and 5, death. Rectal temperature was monitored every 15 min for at least 60 min after challenge. Mice were bled 30 to 90 min after the final challenge, and serum mouse mast cell protease 1 (MCPT-1) levels were determined by ELISA (Invitrogen).

### Statistics

Statistical comparisons were assessed using the Mann-Whitney test or 1-way ANOVA with Dunnett's or Tukey's Test with GraphPad Prism software (version 6; GraphPad Software, La Jolla, Calif). A *P* value of < 0.05 was considered statistically significant. Results presented here are the representatives of at least two independent experiments.

## Results

### Definition of Safety Parameters

**Figure 1:**
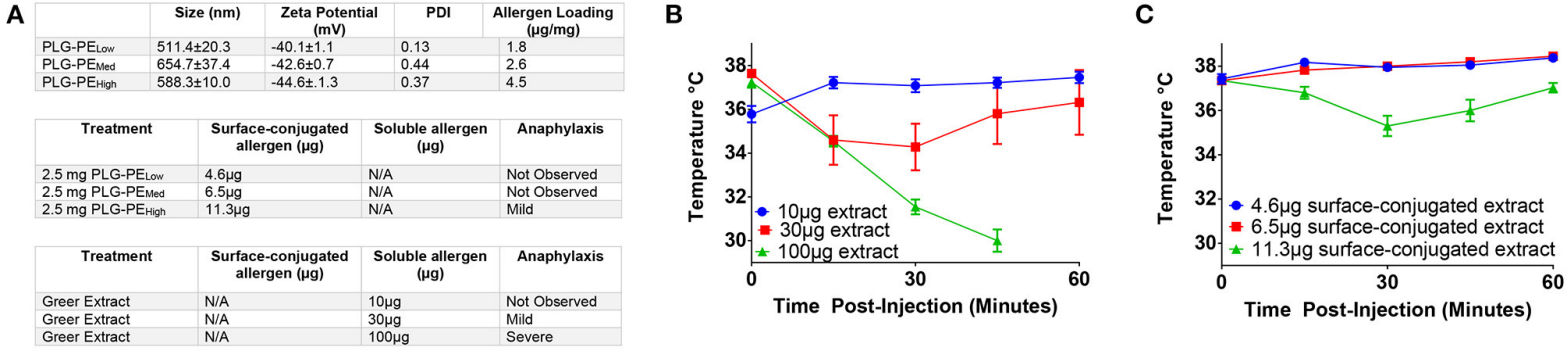
Determination of safety parameters related to delivery of peanut extract via NP. C3H/HeJ mice were sensitized with i.p. injection of peanut and alum at Day−14 and 0. Mice received injection of either peanut extract in PBS or peanut extract surface-conjugated to PLG on Day +7 and were assessed for anaphylaxis as measured by core temperature. **(A)** Antigen loadings (by BCA assay) for each formulation are listed in μg of peanut extract per mg of nanoparticle (μg/mg). Surface-conjugated NP (PLG-PE) were generated with low (1.8 μg/mg), medium (2.6 μg/mg), or high (4.5 μg/mg) loadings of peanut extract. Relative amounts of surface or soluble peanut extract were delivered intravenously and the corresponding observation of anaphylactic response was recorded. **(B)** Core temperatures of mice receiving intravenous injection of peanut extract in PBS (i.e. nanoparticle-free). **(C)** Core temperature of mice receiving intravenous injection of peanut extract surface-conjugated to PLG nanoparticles. Plots display mean measurement ± SEM. *n* = 4–5 per treatment group.

We first sought to identify safety thresholds related to delivery of *exposed* allergen on the surface of NPs. Mice were sensitized with peanut extract and alum and treated with NPs containing various amounts of peanut extract conjugated via carbodiimide chemistry to their surface (Figure 1A). Some delivery systems are susceptible to “burst release” of their cargo, a phenomenon where most or all of the protein content is released immediately upon reconstitution (40). We sought to determine the theoretical maximum amount of tolerable allergen that could be released without inducing a drop in core temperature. Increasing concentrations of peanut extract (PE) in PBS were injected intravenously to imitate the effects of burst release of allergen. A dose of 30 μg of allergen resulted in mild temperature drop while severe adverse reactions were observed at 100 μg of total allergen. A total dose of 10 μg allergen demonstrated no anaphylactic symptoms (Figure 1B). Mice receiving < 11.3 μg of total surface-exposed allergen did not exhibit symptoms of anaphylaxis as evidenced by temperature drop, defining an important parameter for future NP design (Figure 1C). These studies provide key design constraints for the NPs that inform the application of this biomaterial platform and may be generalizable to similar technologies.

### Synthesis and Characterization of Nanoparticles

**Figure 2:**
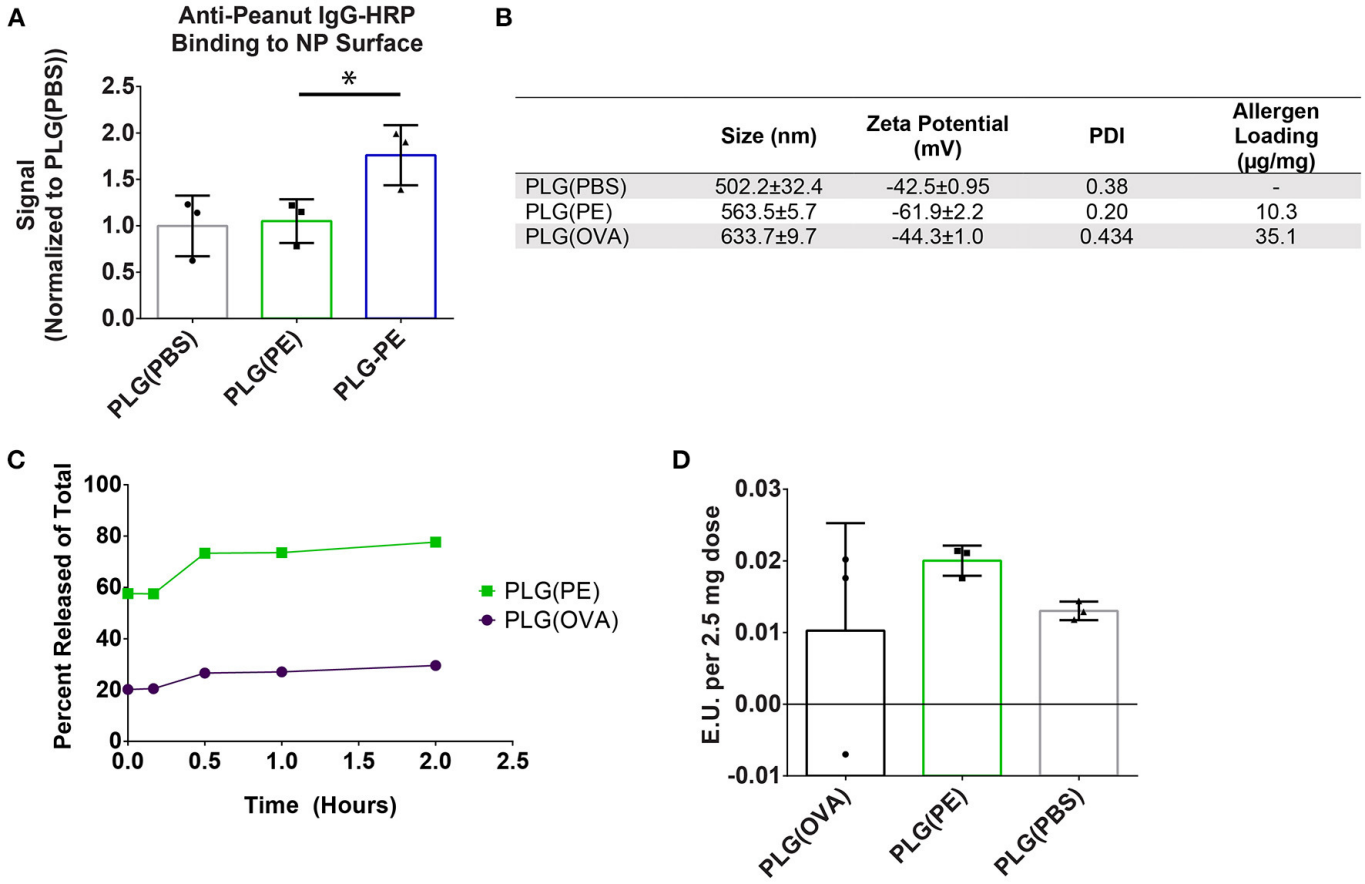
Characterization of PLG NPs used for *in vivo* studies. **(A)** PLG(PE) NPs were confirmed to have low surface expression of peanut extract by measuring amount of Anti-peanut IgG-HRP binding. Total chemiluminescent flux (p/s) was measured using an *in vivo* Imaging System (IVIS) and an exposure time of 1 s, and the signal was normalized to the signal recorded from PLG NPs. **(B)** NP Size, zeta potential, polydispersity index (PDI), and antigen loading (BCA assay, μg antigen per mg NP) of formulations used in subsequent *in vivo* experimentation. **(C)** Antigen release of antigen-containing formulations was measured over 2 h in PBS. **(D)** LAL Endotoxin testing was measured to verify acceptable levels for all formulations utilized. Plots display mean measurement ± SEM. Statistical Significance: 1-way ANOVA with Dunnett's **(A)** multiple comparisons test *p < 0.05.

Based on the safety thresholds (Figure 1), PE or ovalbumin (OVA) were encapsulated within PLG using a water-in-oil-in-water solvent evaporation emulsion method. Anti-peanut antibodies bound more readily to surface conjugated PLG-PE NPs compared to encapsulated PLG(PE) NPs, and no difference in antibody binding to PLG(PE) and PLG(PBS) NPs was observed (Figure 2A). Antigen loading (μg protein per mg NP) was determined by microBCA assay for each formulation (Figure 2B). The protein did not substantially impact NP size, zeta potential, or morphology and all formulations were designed to be within a range of previously-investigated physical characteristics which have shown efficacy in other models (15, 16). Antigen release over 2 h demonstrated a burst release of approximately 60% to 80% of the PE from PLG(PE) between 15 min and 2 h (Figure 2C). Endotoxin was measured and confirmed to be below the acceptable limits established for Sterile Water for Injection (< 0.25 EU/mL) for each formulation at our 2.5 mg, 100 μL dose (Figure 2D) (41, 42).

### PLG(PE) Administration Is Well-Tolerated in Pre-sensitized Mice

**Figure 3:**
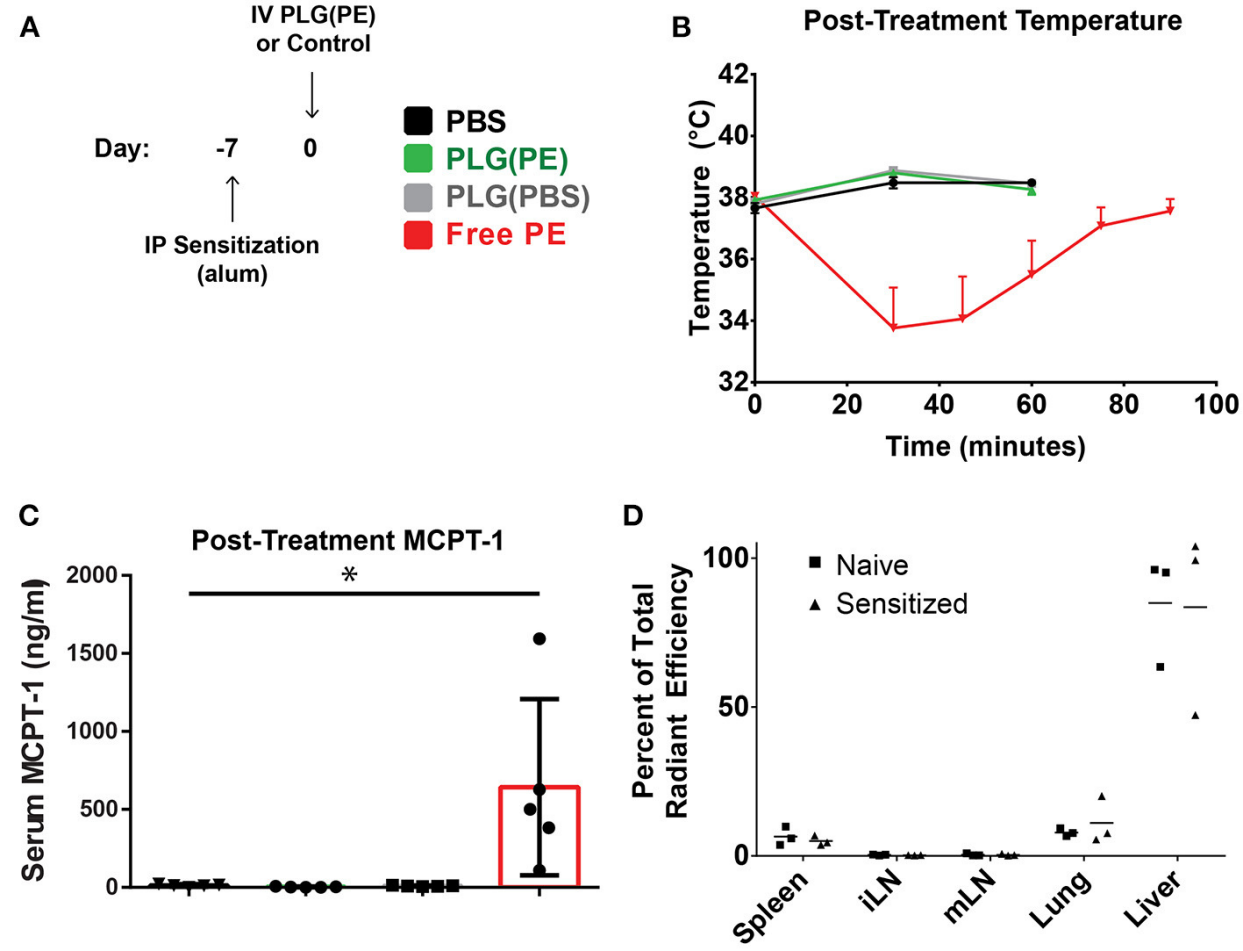
PLG(PE) is well-tolerized in pre-sensitized mice. C3H/HeJ mice received i.p. injection of alum and peanut extract on Day−7 relative to NP injection **(A)**. Mice were treated on Day 0 with PLG(PE) NPs or controls and assessed for anaphylactic response to therapy. Intravenous injection of PLG(PE), PBS, or PLG(PBS) were well-tolerated, while injection of an equivalent total dose (25.75 μg) of soluble peanut extract resulted in anaphylactic temperature drop (B) and serum release of MCPT-1 **(B)** (*n* = 5). **(C)** Cyanine5.5-labeled, peanut extract-loaded NPs [PLG(PE, Cy5.5)] were injected intravenously. Twenty four hours post-injection, spleens, inguinal lymph nodes, mesenteric lymph nodes, lungs, and livers were collected and imaged by IVIS to determine relative organ distribution in naïve and pre-sensitized mice, presented as percent of total radiant efficiency **(D)**. Plots display mean measurement ± SEM Statistical Significance: 1-way ANOVA with Tukey's multiple comparisons test: *P < 0.05.

We next investigated whether PLG(PE) would be well-tolerated in mice with established food allergy, confirming the safety of these i.v.-administered allergen-encapsulated NPs in a therapeutic setting. Mice were sensitized by i.p. administration of peanut extract adsorbed on alum as previously described (34). After sensitization, mice were administered PBS, PLG(PBS) NPs, PLG(PE) NPs containing 25.75 μg of total peanut extract, or an equivalent dose (25.75 μg) of soluble PE in PBS (at an equivalent volume). Mice treated with PBS, PLG(PBS), or PLG(PE) NPs (Figure 3A) did not show evidence of anaphylaxis as measured by core body temperature (Figure 3B) or MCPT-1 release in serum (Figure 3C); however, an equivalent dose (25.75 μg) of soluble PE in PBS did cause significant allergic reactions, as evidenced by temperature decrease and MCPT-1 release, demonstrating the ability of PLG(PE) to mask allergen to avoid reactivity in allergic individuals.

We subsequently investigated the impact that sensitization has on biodistribution, which may provide insight into the mechanisms through which NPs promote improved clinical outcomes. The NP platform designed to encapsulate antigen may still display some level of surface antigen, and serum allergen-specific antibodies within pre-sensitized individuals could bind these NPs upon administration and influence their biodistribution and thus their subsequent function. PLG(PE) NPs fluorescently tagged with Cy5.5 were intravenously administered into mice that were pre-sensitized by i.p. administration of PE adsorbed on alum (15, 16). Whole animal fluorescence imaging quantified the percent of total radiant efficiency contributed by each organ (Figure 3D), with most NPs localizing to the liver, followed by the lung and spleen. The relative distribution of NPs between sensitized and naïve animals was not significantly different (*P* > 0.05).

### Prophylactic Administration of PLG(PE) Prevents Sensitization to Peanut Antigen

**Figure 4:**
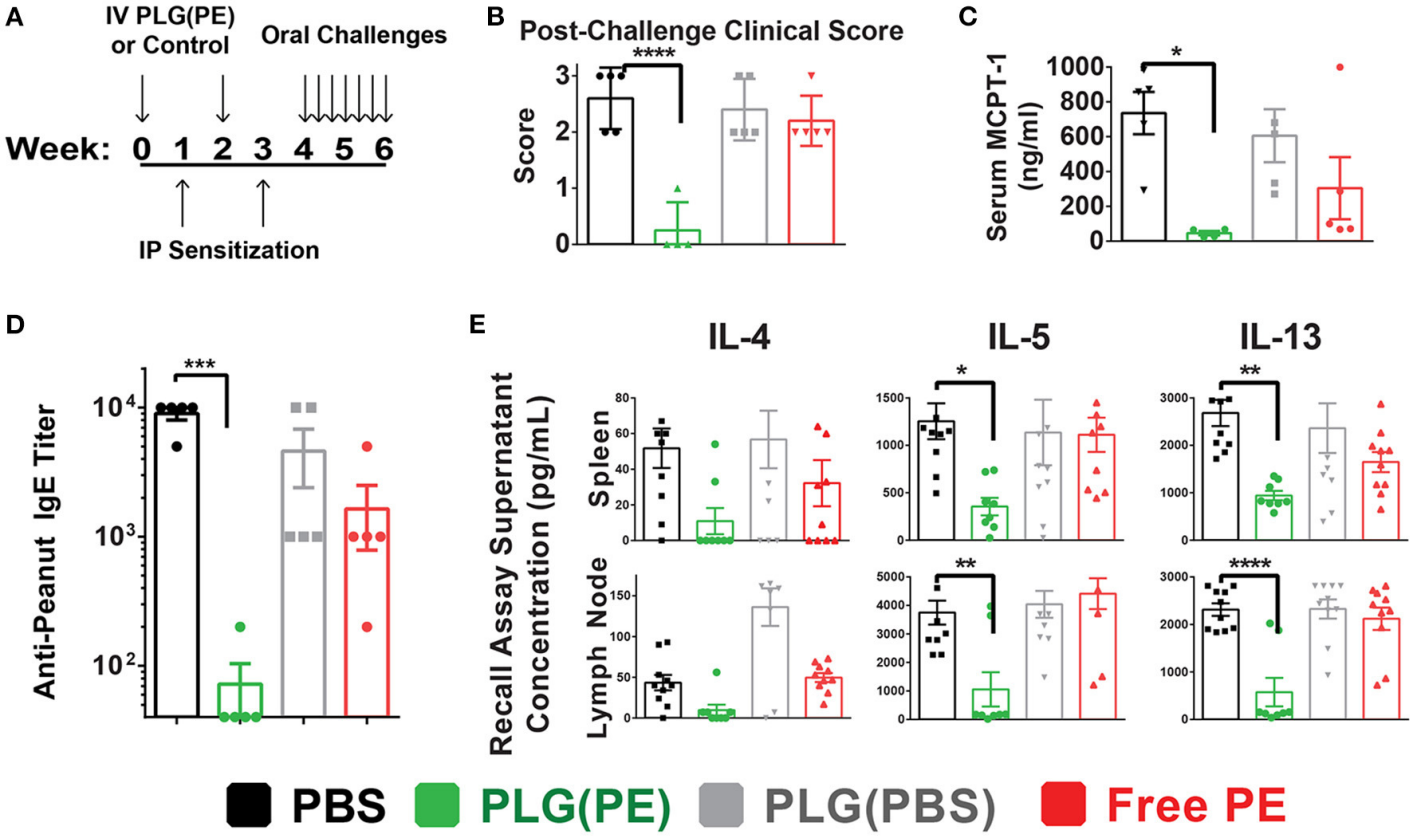
PLG(PE) provides prophylactic protection from peanut allergy. C3H/HeJ mice were sensitized by i.p. injection of alum and peanut extract on Day 7 and 21 after receiving treatment or control on days 0 and 14. Seven intragastric challenges took place over days 28 through 42. Data represent the response to the 7th intragastric challenge. Schematic of experimental timeline **(A)**. Clinical anaphylaxis score was characterized **(B)** and serum MCPT-1 levels determined by ELISA **(C)**. Serum peanut-specific IgE levels were characterized **(D)** and cytokine secretion in culture supernatants of recall assays were measured by Luminex multiplex **(E)**. *n* = 5 per group. Plots display mean measurement ± SEM. Statistical significance determined with 1-way ANOVA with Dunnett's multiple comparisons test: *P < 0.05, **P < 0.01, ***P < 0.005, ****P < 0.001.

Prophylactic administration of PLG(PE) was studied to determine whether nanoparticles would induce a level of tolerance sufficient to prevent sensitization to peanut (Figure 4A). Mice that were sensitized after treatment with PLG(PBS) NPs or PBS displayed significant reactions to intragastric peanut challenge as measured by Clinical Score (Figure 4B) and serum MCPT-1 after intragastric challenge, while reactivity was significantly suppressed in mice that were prophylactically treated with PLG(PE) (Figure 4C).

The mechanism by which sensitization was prevented was assessed in part through serum anti-peanut IgE and allergic cytokine production by mesenteric lymph node and splenic cells following stimulation with peanut. PLG(PE) significantly reduced production of serum anti-peanut IgE, with expression below detectable threshold in 80% of mice (Figure 4D). Analysis of the response to peanut in recall assays of splenic and mesenteric lymph node cells showed a significant reduction in secretion of Th2 cytokines, indicating suppression of allergic immune responses (Figure 4E). These results demonstrate that effects of PLG(PE) were sufficiently robust to prevent sensitization efforts in a Th2-mediated model of peanut allergy.

### Therapeutic Administration of PLG(PE) Reduces Reactivity to Intragastric Peanut Challenge

**Figure 5:**
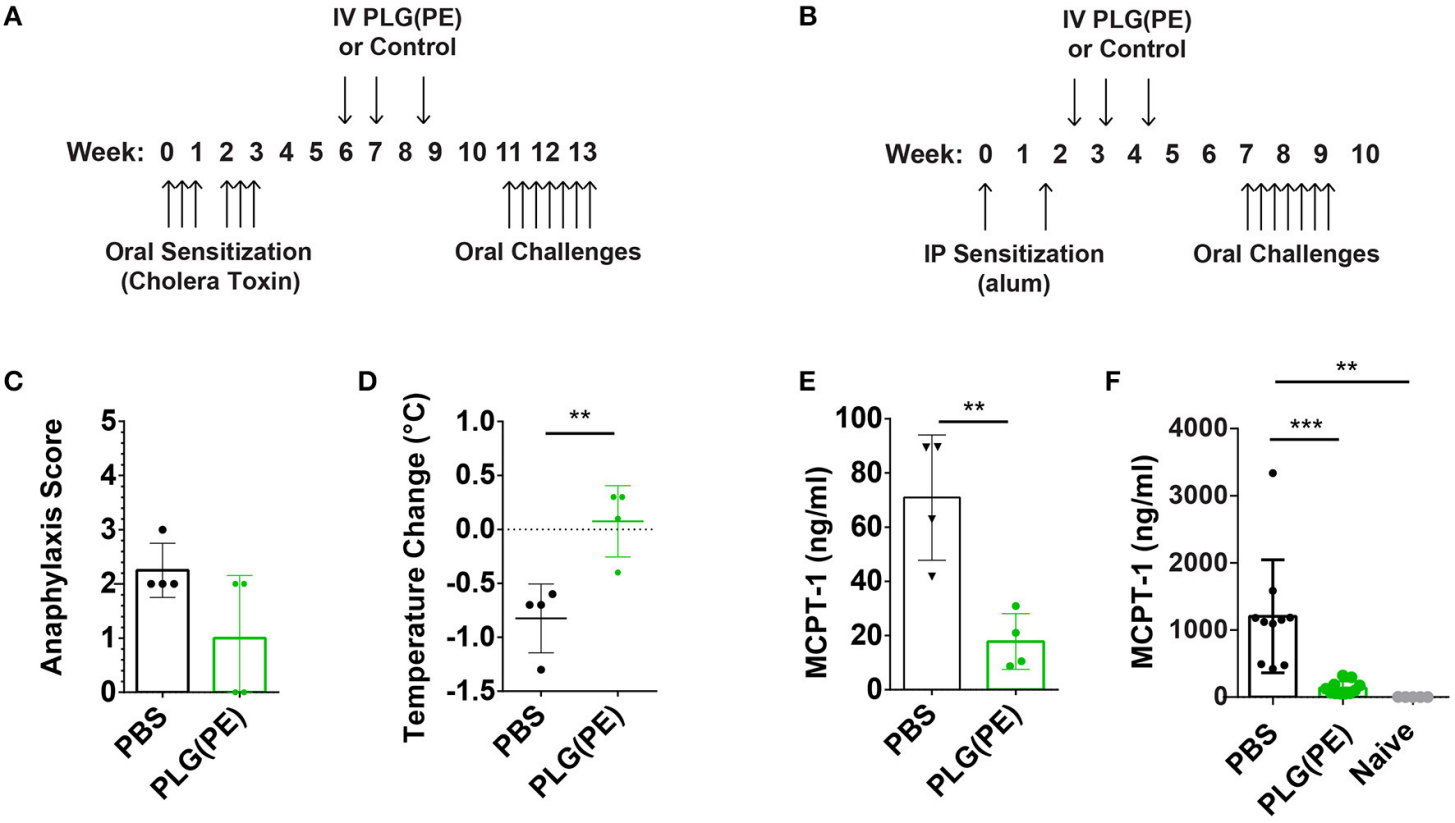
Therapeutic efficacy of PLG(PE) in multiple murine models of peanut allergy. Mice were sensitized with cholera toxin (*n* = 4 per group) beginning on Day 0, were treated with PLG(PE) on days 42, 49, and 59, and received intragastric challenge over days 77 through 91 **(A)**. Symptoms of anaphylaxis and body temperature were recorded every 15 min for at least 1 h after the 7th challenge, and data represent the maximum change in temperature. PLG(PE) trended toward protection against anaphylactic symptoms **(C)** and significantly reduced the degree of temperature drop **(D)** as well as serum MCPT-1 **(E)**. Results were confirmed by measuring serum MCPT-1 in alum-sensitization. **(B)** Mice were sensitized with alum on Days 0 and 14, treated with PLG(PE) on Days 21, 28, and 38, and received intragastric challenges over days 49 through 63. PLG(PE) provided protection from serum MCPT-1 release, indicating clinical protection from anaphylaxis **(F)**. Plots display mean measurement ± SEM. Statistical significance determined by Mann-Whitney **(C–E)** or One-Way ANOVA with Dunnett's multiple comparisons test **(F)**. **P < 0.01, ***P < 0.005.

We next investigated the efficacy of therapeutically administered PLG(PE) in mice with established peanut allergy. Mice were sensitized intragastrically with peanut and cholera toxin and received three doses of PLG(PE) (Figure 5A). Treated mice exhibited a reduced (though not statistically significant) reduction in anaphylactic symptoms (Figure 5C), a significantly smaller drop in core temperature (Figure 5D), and a significant reduction in serum MCPT-1 (Figure 5E). These studies were repeated in an alum-induced model of food allergy (Figure 5B) that is associated with more severe mast cell degranulation and demonstrated a similar reduction in MCPT-1 in PLG(PE) treated mice (Figure 5F). These results indicate that PLG(PE) can significantly prevent mast cell degranulation that occurs upon allergen challenge and initiates the allergic cascade.

### Therapeutic Administration of PLG(PE) Is Associated With Significant Reduction in Cytokine Production in an *ex vivo* Recall Assay

**Figure 6:**
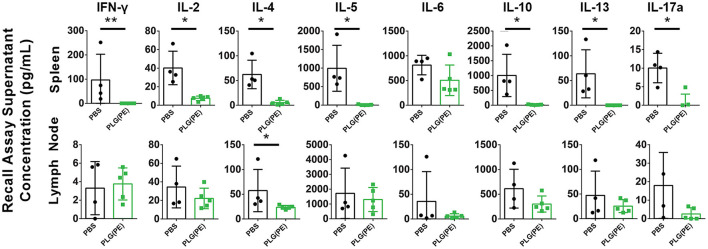
Investigation of mechanisms involved with therapeutic efficacy of PLG(PE) in alum-sensitized mice. Mice were sensitized with alum on Days 0 and 14 and treated with PLG(PE) on Days 21, 28, and 38. Cytokine production from spleen or mesenteric lymph node cells cultured with crude peanut extract were measured by Luminex multiplex assay. Statistical significance determined by Mann-Whitney test: *P < 0.05, **P < 0.01.

Therapeutic efficacy of PLG NPs in murine models of Th1/17 disease and airway allergy has been associated with reduction in antigen-specific cytokine production, suggesting the induction of tolerance, or non-responsiveness, to disease-relevant antigens (15–18). *Ex vivo* analysis of the response of spleen and lymph node cells from control vs. PLG(PE) treated mice demonstrated suppression of peanut-specific cytokine production following PLG(PE) treatment. In particular, in spleen cell cultures, IFN-γ, IL-2, IL-4, IL-5, IL-10, IL-13 and IL-17a production were all significantly reduced in PLG(PE) treated mice relative to control PBS-treated mice, and IL-6 showed a similar trend. Similar results were observed in mesenteric lymph node cell cultures, though only IL-4 production was significantly different. PLG(PE) treated mice did not have increased production of any cytokine that was tested. Suppression across a wide variety of cytokines stereotypically produced by T helper subsets suggests that the mechanism of action is not dependent on a skewing of T cell phenotypes but is rather the result of a broad suppression of the cellular immune response to peanut antigens (Figure 6).

## Discussion

Food allergy is a life-threatening disease that represents a considerable quality of life reduction for patients and a large economic burden for society (7, 34). While AIT is available for some patients, these treatments carry high risk of anaphylaxis, require months or years of treatment, and have not demonstrated long-term efficacy in most patients without continued daily administration (10, 43). Despite advances in recent years, a significant need remains for the development of novel therapeutics for food allergy. Herein, we investigate a PLG NP platform for efficacy in prophylactic and therapeutic treatment of peanut allergy. Delivery of allergen in NPs would provide multiple benefits over AIT including targeted delivery to relevant antigen presenting cells, reduced epitope exposure preventing undesired immune recognition, and the potential to achieve efficacy with fewer doses, each carrying a lower amount of allergen. In these studies, mice received doses of ~1.3 mg peanut/kg. The Palforzia daily maintenance dose is a constant 300 mg dose, regardless of patient mass. Collectively, NP delivery represents a reduction in the total exposure to allergen (44).

Intravenous delivery of allergens for treatment of food allergy is not frequently attempted, although literature suggests this route may be effective in inducing high-zone tolerance (45–47). By encapsulating allergen or interfering with epitope availability via surface conjugation, risk of anaphylaxis is expected to be substantially lower than delivery of soluble allergen alone. Two commonly used methods of antigen incorporation in polymeric NP formulations involve encapsulation and surface-conjugation. Encapsulation may reduce the risk of surface-exposed allergen triggering degranulation of effector mast cells in pre-sensitized individuals; however, a risk of anaphylaxis exists that is related to the burst release of contents upon reconstitution or injection. Alternatively, conjugation of allergen to NPs might alter IgE binding and reduce effector cell activation; however, if those sites are not sufficiently disrupted, surface presentation of allergen may carry a risk of anaphylaxis. Interestingly, despite a lower total quantity of allergen exposed for immune recognition than the NP-free peanut extract, a similar degree of temperature reduction was observed, which may result from several factors: quantification of protein against complex backgrounds can limit the accuracy of measurement (31). Alternatively, oligomerization or repetition of B cell epitopes can increase cellular activation. Concentration of allergen on the surface of NPs might exceed a threshold of activation that would not be achieved by a more diluted allergen in circulation. The mechanisms by which NPs can activate effector cells is an active area of research in food allergy.

To improve NP tolerability, we utilized peanut-encapsulated PLG(PE) NPs in subsequent studies based on the observed reactivity toward surface-conjugated NPs in Figure 1. The formulation would ideally have minimal levels of surface-bound peanut extract and would not release allergen until the NP is internalized. Antibody staining confirmed that PLG(PE) had low levels of surface-expressed allergen. PLG(PE) measurements were nearly identical to the background antibody binding of similarly treated PLG(PBS) NPs fabricated without peanut, while binding to surface-conjugated PLG-PE was significantly greater. The PLG(PE) used herein contained 10.3 μg peanut extract per mg of NP, delivering a total of <30 μg. Nevertheless, the release profile of PLG(PE) was measured, with release of 64% (i.e., 16.5 μg for a 2.5 mg dose) shortly after incubation in PBS (40, 48). Delivery of 2.5 mg of PLG(PE) (25.75 μg of total protein) and controls [PBS or PLG(PBS)] demonstrated the NPs were well-tolerated in pre-sensitized mice, while an equivalent dose of peanut extract in PBS caused reactivity. Despite the relatively high burst release from the present PLG(PE) formulation, these NPs were efficacious in both therapeutic and prophylactic experiments.

In previous studies, incorporation of antigen in 500 nm diameter NPs facilitates delivery of allergen to relevant APCs and presentation to T cells, which is expected to afford improved efficiency over injection of soluble allergen alone (15, 16, 49, 50). Despite the reduction in a variety of cytokines implicated in allergic disease, peanut-specific antibody titers were not significantly altered with therapy (Supplementary Figure 1). These results suggest a predominant T cell-mediated mechanism of action and are consistent with previous studies performed with this NP platform in models of Th1/17-driven disease (15, 16, 51). Based on the size of these NPs, we expected similar organ-level distribution; however, we considered that, in a pre-sensitized mouse, peanut-specific immunoglobulins could conceivably bind PLG(PE) and impact the distribution. Nevertheless, the biodistribution of PLG(PE) NPs was not different between naïve and peanut sensitized mice. Dose escalation of PLG(PE) in a model of delayed type hypersensitivity significantly inhibited challenge response for all concentrations higher than 0.05 mg/dose, with similar efficacy in the 0.75, 1.0, and 2.5 mg/dose groups (Supplementary Figure 2). These results demonstrate an ability to reduce reactivity to peanut in a dose-dependent fashion in an additional model of antigen-specific T cell activation. These data, as well as the measurement of anti-peanut IgG binding to NPs, support the hypothesis that encapsulation of allergen can reduce the risk of recognition of NPs by effector cells.

Collectively, our studies demonstrate that peanut-encapsulated [PLG(PE)] NPs are well-tolerated and efficacious at inducing tolerance to prevent sensitization and reducing allergic reactivity in previously sensitized mice. Importantly, this platform demonstrated safety and efficacy in both prophylactic and therapeutic settings of two commonly used murine models of peanut allergy. The PLG NPs used in these studies demonstrated antigen-specific broad suppression of Th2 cell responses that was not associated with skewing of T cell phenotypes toward a Th1/17 path. Interestingly, despite these observations there was not a reduction in antigen-specific antibodies observed in the serum, suggesting that this therapy may not completely regulate plasma cells in the observed timeframe. The long half-life of immunoglobulins may require additional time to note any potential changes to the antibody repertoire induced by this therapy. Additionally, quantification of antibodies circulating in the serum may not directly reflect the amount of antibodies bound to effector cells via Fc receptors. A role for modulation of antibodies in the tissues or bound to effector cells cannot be excluded by our current data. Our studies suggest that protective effects conferred by PLG(PE) NPs are more similar to tolerance, or a lack of responsiveness to allergen, than a general skewing away from a Th2 phenotype.

These experiments demonstrate the ability of peanut-encapsulating PLG NPs to induce a phenotype consistent with tolerance in peanut-sensitized mice. The well-tolerated delivery of μg-quantities of allergen across only 2–3 doses achieves therapeutic outcomes in multiple clinically relevant models of peanut allergy. Current immunotherapies under evaluation for food allergy therapy require months or years of administration with considerable risk of anaphylaxis and limited evidence of long-term clinical unresponsiveness (10, 34, 52, 53). These results represent a strategy for food allergy therapeutics that may prove to be less burdensome and more representative of immunological tolerance than current AIT options (9, 34, 54).

## Data Availability Statement

The raw data supporting the conclusions of this article will be made available by the authors, without undue reservation.

## Ethics Statement

The animal study was reviewed and approved by University of Michigan Institutional Animal Care and Use Committee.

## Author Contributions

KH and JP primarily conceptualized the experiments included in this manuscript. MS, JL, KJ, HT, JP, and LR also conducted and analyzed the experiments. The paper was initially drafted by KH with input from MS, LR, SM, JP, LS, and JO'K. All authors contributed to the article and approved the submitted version.

## Funding

MS is supported by NIH grant T32GM007863. Research reported in this publication was supported by the National Institute of Allergy and Infectious Diseases of the National Institutes of Health under award numbers R01AI148076 and R01AI155678.

## Conflict of Interest

LS, JP, and SM have financial interests in COUR Pharmaceuticals Development Co. The remaining authors declare that the research was conducted in the absence of any commercial or financial relationships that could be construed as a potential conflict of interest.

## Publisher's Note

All claims expressed in this article are solely those of the authors and do not necessarily represent those of their affiliated organizations, or those of the publisher, the editors and the reviewers. Any product that may be evaluated in this article, or claim that may be made by its manufacturer, is not guaranteed or endorsed by the publisher.
