# Supplementary material for: Masked Delivery of Allergen in Nanoparticles Safely Attenuates Anaphylactic Response in Murine Models of Peanut Allergy
Source: Front Allergy. 2022 Feb 7;3:829605. doi: 10.3389/falgy.2022.829605 (PMC8974743; doi:10.3389/falgy.2022.829605)
Supplement: Supplementary file 1 [file Data_Sheet_1.docx]

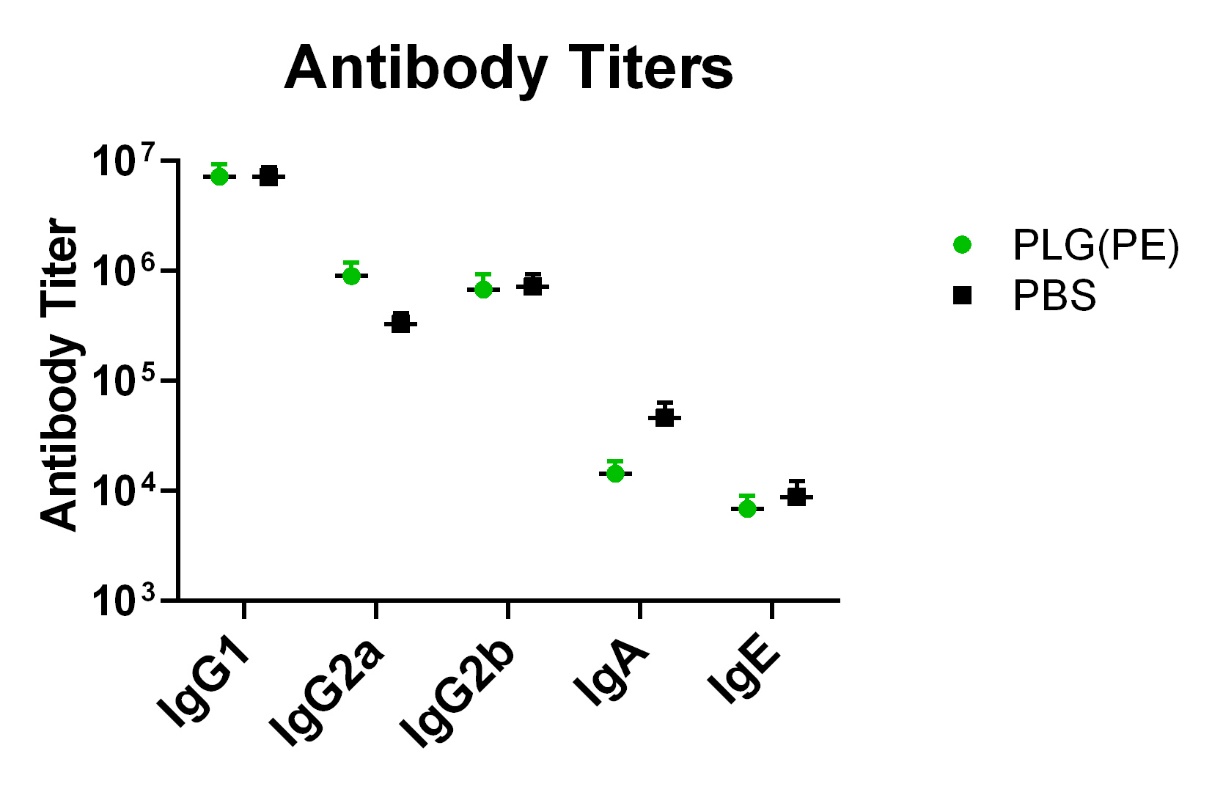


**Supplementary Figure 1**: C3H/HeJ mice were sensitized with alum on Days 0 and 14 and treated with PLG(PE) or PBS on Days 21, 28, and 38. Mice were challenged seven times over days 49 through 63 and 7 additional times over days 165 through 179. On day 179 plasma were collected and analyzed for anti-peanut IgG1, IgG2a, IgG2b, IgA, and IgE via ELISA. Results are given as mean +/- SEM; *n*=9 for PLG(PE), *n*=10 for PBS.


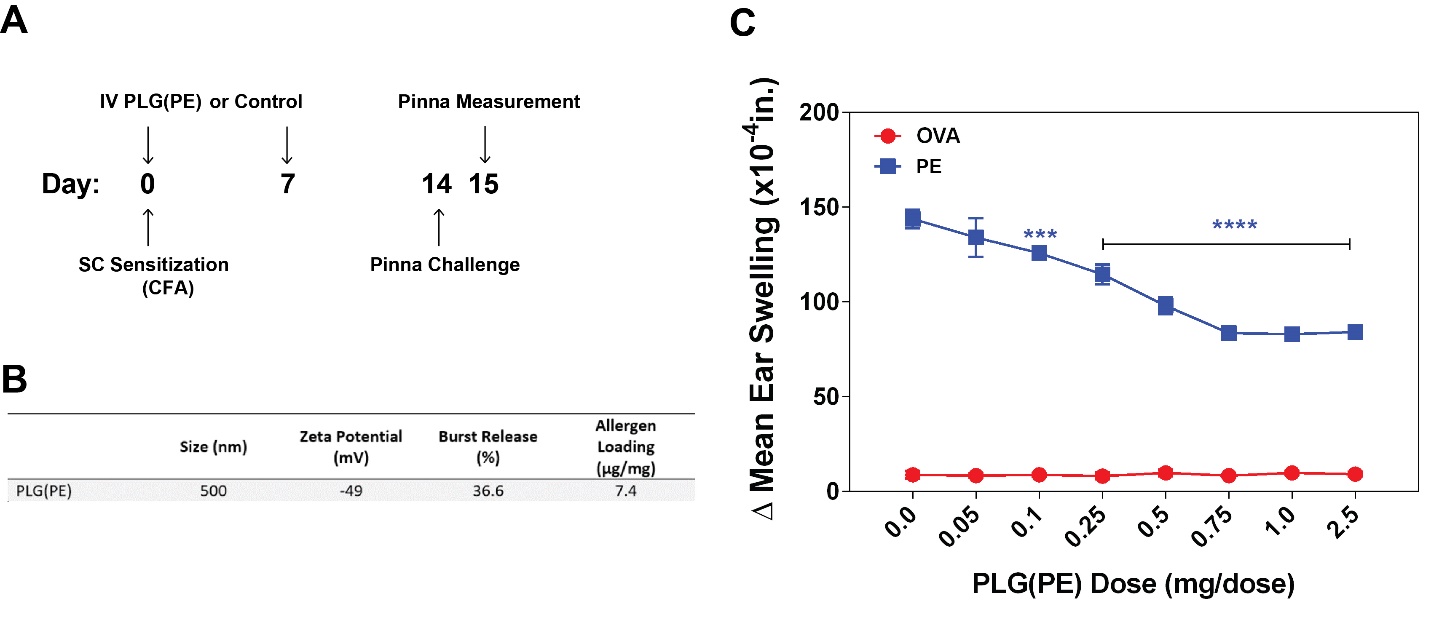


**Supplementary Figure 2**: PLG(PE) was administered to Purified Peanut Extract (PPE)/CFA primed C57BL/6 mice at 0.05, 0.1, 0.25, 0.5, 0.75, 1, and 2.5 mg/dose in 200 μL on Day 0 and Day 7 relative to CFA administration (A). On Day 14 post CFA administration, the baseline pinna thickness of both ears of each mouse was measured. On the same day, mice were intradermally challenged with 10 μg (10 μL injection volume) OVA in the left ear and 10 μg (10 μL injection volume) PPE in the right ear. The pinna thickness of both ears of each mouse was evaluated 24 hours post challenge, and the delta pinna thickness was calculated. Antigen loadings (by CBQCA assay) for formulation are listed in µg of peanut extract per mg of nanoparticle (µg/mg) (B). Delayed Type Hypersensitivity dose titration of PLG(PE). Results are given as mean ± SEM; *n* = 5 for each group, and one representative experiment of three is presented. Statistical significance determined by One-Way ANOVA with Dunnet’s multiple comparisons test in comparison to 0 mg/dose treatment per group (C).
